# Review: Vaccines and Vaccination against Lumpy Skin Disease

**DOI:** 10.3390/vaccines9101136

**Published:** 2021-10-06

**Authors:** Eeva Tuppurainen, Klaas Dietze, Janika Wolff, Hannes Bergmann, Daniel Beltran-Alcrudo, Anna Fahrion, Charles Euloge Lamien, Frank Busch, Carola Sauter-Louis, Franz J. Conraths, Kris De Clercq, Bernd Hoffmann, Sascha Knauf

**Affiliations:** 1 Institute of International Animal Health/One Health, Friedrich-Loeffler-Institut, Federal Research Institute for Animal Health, Südufer 10, D-17493 Greifswald-Insel Riems, Germany; Klaas.Dietze@fli.de (K.D.); Anna.Fahrion@fli.de (A.F.); Frank.Busch@fli.de (F.B.); Sascha.Knauf@fli.de (S.K.); 2 Institute of Diagnostic Virology, Friedrich-Loeffler-Institut, Federal Research Institute for Animal Health, Südufer 10, D-17493 Greifswald-Insel Riems, Germany; Janika.Wolff@fli.de (J.W.); Bernd.Hoffmann@fli.de (B.H.); 3 Institute of Epidemiology, Friedrich-Loeffler-Institut, Federal Research Institute for Animal Health, Südufer 10, D-17493 Greifswald-Insel Riems, Germany; Hannes.Bergmann@fli.de (H.B.); Carola.Sauter-Louis@fli.de (C.S.-L.); Franz.Conraths@fli.de (F.J.C.); 4 Regional Office for Europe and Central Asia, Food and Agriculture Organization, 20 Kalman Imre utca, H-1054 Budapest, Hungary; Daniel.BeltranAlcrudo@fao.org; 5 FAO/IAEA Division of Nuclear Techniques in Food and Agriculture, Department of Nuclear Sciences and Applications, International Atomic Energy Agency (IAEA), Friedenstrasse 1, A-2444 Seibersdorf, Austria; C.Lamien@iaea.org; 6 Unit of Exotic and Particular Diseases, Scientific Directorate Infectious Diseases in Animals, Sciensano, Groeselenberg 99, B-1180 Brussels, Belgium; kris.declercq@sciensano.be

**Keywords:** capripoxvirus, lumpy skin disease, cattle, LSD, immunization, vaccination

## Abstract

The geographical distribution of lumpy skin disease (LSD), an economically important cattle disease caused by a capripoxvirus, has reached an unprecedented extent. Vaccination is the only way to prevent the spread of the infection in endemic and newly affected regions. Yet, in the event of an outbreak, selection of the best vaccine is a major challenge for veterinary authorities and farmers. Decision makers need sound scientific information to support their decisions and subsequent actions. The available vaccine products vary in terms of quality, efficacy, safety, side effects, and price. The pros and cons of different types of live attenuated and inactivated vaccines, vaccination strategies, and associated risks are discussed. Seroconversion, which typically follows vaccination, places specific demands on the tools and methods used to evaluate the effectiveness of the LSD vaccination campaigns in the field. We aimed to give a comprehensive update on available vaccines and vaccination against LSD, to better prepare affected and at-risk countries to control LSD and ensure the safe trade of cattle.

## 1. Introduction

**Figure 1:**
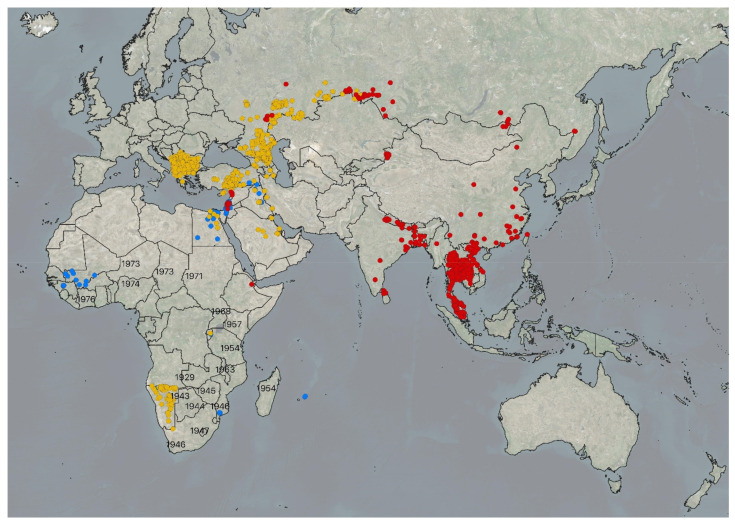
Geographical extent of lumpy skin disease outbreaks reported to EMPRES Global Animal Disease Information System of the Food and Agriculture Organization (https://empres-i.review.fao.org/#/, last accessed 23 September 2021) between January 2006 and May 2021. Outbreaks marked with blue dots were reported in 2006–2013, yellow in 2014–2018, and red in 2019–2021. LSD is endemic across the African continent, except in Algeria, Morocco, Libya, and Tunisia. Historical data from Africa were taken from Woods (1990) [8]. Black numbers indicate the year of the first recorded LSD outbreaks in the respective country.

Large-scale regional vaccination of cattle and Asian water buffalos (*Bubalus bubalis*) is the most effective tool to halt the spread of lumpy skin disease (LSD) and to minimize cattle production losses caused by outbreaks [1]. The disease has hampered cattle and domestic buffalo production throughout most of the African continent, the Middle East, the Balkans, the Caucasus, and the Russian Federation [2]. Since 2019, LSD has reached some of the major cattle producing and trading countries across Asia, for example India [3], the Republic of China [4], Myanmar [2], Bangladesh [5], Vietnam [6], and, most recently, between May and September 2021, Cambodia, Malaysia, Lao People’s Democratic Republic, and Mongolia (OIE WAHIS). Figure 1 illustrates the distribution of the disease in September 2021. Successful eradication of the disease in the Balkans [7] demonstrates that LSD can be effectively controlled when there is a regional willingness to harmonize measures required to prevent the spread of the disease, including vaccines, vaccination campaigns, restrictions relating to cattle movements and trade, a feasible stamping out policy, disinfection, and vector control.

Lumpy skin disease virus (LSDV) belongs to the genus *Capripoxvirus* of the family *Poxviridae* [9]. Infected animals typically show fever, poor general body conditions, reduced feed and water uptake, lowered milk production, enlarged lymph nodes, and characteristic skin nodules. The number of the lesions may vary from a few in mild cases, to multiple lesions, covering the entire body in severely infected individuals. In addition, necrotic plaques may appear in the mucous membranes of the oral and nasal cavities, causing purulent or mucopurulent nasal discharge and excessive salivation. Moreover, ulcerative lesions may appear in the cornea of one or both eyes, leading to restricted vision, and even to blindness. Severe cases may show characteristic lesions throughout the entire digestive and respiratory tracts and on the surface of almost any internal organ [10,11].

Transboundary LSD is categorized as a notifiable disease by the World Organization for Animal Health (OIE) and has a substantial economic impact on the cattle industry due to decreased milk and meat production, abortions, fertility problems in males and females, damaged cattle skins, and, ultimately, the death of severely affected animals. Indirect losses are caused by national and international cattle movement and trade restrictions. The high costs of vaccines and vaccination, diagnostic services, disinfection of facilities, and treatment of severely infected animals add to the costs [2,12]. A short-lived drop in milk yield is a common observation in cattle vaccinated for the first time [13].

LSD is a vector-borne disease, mechanically transmitted by blood-feeding mosquitos, biting flies [14,15], and some tick species [16,17,18]. The mechanical transmission mode (transmission of the virus does not depend on the capability of the virus to multiply inside vectors) is likely to allow the successful spread of the virus by any biting arthropod species if it prefers cattle or domestic buffalos, feeds frequently, and changes the host between the blood meals. The long-distance spread of LSDV has been associated with the transport of live cattle or buffalos.

Direct contact [19] and seminal [20] transmission have been demonstrated experimentally, along with intrauterine transmission in the field [21]. Early observations have also supported the transmission of the virus via contaminated feed and water [11].

During an outbreak, large-scale immunization of bovines is the most effective control measure when combined with bovine movement restrictions [1]. In almost all currently affected regions, the restriction of cattle or domestic buffalo movements face serious challenges, particularly in regions that lack the census data of cattle and buffalo populations, individual animal identification systems, and associated central record keeping, including movements of bovines. Farmers constitute the first line of defense against this highly contagious transboundary disease. Small-scale cattle and buffalo trade across the borders occurs in many currently affected regions, despite not being authorized [2]. Moreover, farmers may hesitate to report suspected LSD outbreaks to the veterinary authorities due to fear of consequent negative impacts, such as ban of cattle movements and trade. As a result, delayed detection and reporting of outbreaks will hamper a successful control of LSD. Traditional cattle farming practices, such as the seasonal transport of animals to summer and winter pastures are reported risk factors for LSD [22,23]. However, a sudden halt of these practices is likely to lead to animal welfare problems if no alternative feeding system is in place or if there is insufficient space to keep the additional animals. Some religious and cultural festivities, comprising animal sacrifice, increase the number of cattle, sheep, and goats transported prior to the festive season and are clearly associated with the spread of LSD [24].

In order to halt the spread of LSD by vectors in particular, culling of all susceptible animals that have been exposed to the infection, or at least those showing clinical signs, is a generally recommended control measure for LSD [25]. However, in many resource-limited countries, this measure may not be affordable or feasible. Moreover, the killing of cattle or other affected animals may not be permitted by law or due to religious and traditional reasons and, consequently, stamping out cannot be accomplished [2]. All the above-discussed facts highlight the importance of swift mass vaccinations of cattle and water buffalos, using a high-quality vaccine with proven efficacy against the virus. We review the currently available vaccines against LSD, as well as vaccination strategies in countries where LSD is endemic or in countries that face a high risk of LSD introduction.

## 2. Production, Quality, Transport, Storage, and Handling of the LSD Vaccines

Currently, most commercially available vaccines against LSD are live attenuated vaccines based on a LSDV strain, sheeppox virus (SPPV), or goatpox virus (GTPV) (Table 1). The first inactivated vaccine has recently entered the market. The protection provided by a vaccine against LSDV depends on the seed virus, the level of attenuation, the volume of the dosage, and the titer of the attenuated vaccine virus in the final product. The quality of a vaccine product is of major importance to farmers and the welfare of cattle. Therefore, vaccines should be produced according to the good manufacturing practices (GMP). The guidelines for the production and control of immunological veterinary medicinal products have been published, for example by the European Medicines Agency (https://www.ema.europa.eu/en/documents/scientific-guideline/guideline-requirements-production-control-immunological-veterinary-medicinal-products_en-0.pdf; last visited in 31 May 2021). In addition, the OIE has defined the technical requirements for the vaccines used against LSD [26,27,28].

A good-quality vaccine, as well as its seed lots of virus, continuous cell lines, or primary cell culture batches and biologicals used for virus growth, must be free of extraneous viruses, mycoplasmas, other bacteria, fungi, protozoa, and rickettsia [26,27].

The vaccine seed virus must be characterized using molecular tools and the origin and passage history should be well documented. The use of an uncharacterized live vaccine may, in the worst-case scenario, lead to infection of the vaccinated animals with unwanted extraneous pathogens. An over-attenuated vaccine loses its ability to protect cattle against LSDV, causing subsequent economic losses for farmers. In contrast to over-attenuation, the use of under-attenuated vaccines may lead to an unacceptably high number of post-vaccination adverse reactions [29]. It is essential to regularly check, using molecular tools, whether the vaccine contains the correct capripoxvirus genotype and strain, as well as examine subpopulations of viruses in the vaccine. When an LSD outbreak is ongoing and mass vaccination campaigns are carried out quickly, vaccination of already infected animals may increase the risk of recombination of the vaccine and field strains [30]. Furthermore, due to the shortcomings of vaccines derived from an LSDV strain, Kenyan cattle-1 (KS1), many countries tend to use the Neethling strain vaccine for cattle while keeping the KS1-derived vaccines for the immunization of small ruminants. Hence, some vaccine producers in LSDV-endemic countries offer both types of live attenuated LSDV vaccines. However, producing those two vaccines on the same production lines creates a risk of cross-contamination between the two vaccine viruses.

LSD vaccines must be safe to use in all age groups, both sexes, and all breeds and bovine species [28]. Since vaccination can be associated with the appearance of side effects, a full description of adverse reactions needs to be included in the package insert. This prevents confusion of vaccine-associated side effects with wild-type infections. It may also reduce general concerns farmers may have regarding LSD vaccination [31] and provide advice on the measures to be carried out if adverse effects are detected. Vaccine producers should also define the guidelines for the safe use of the vaccine in case of cold chain breakdowns or after reconstitution of a vial of a freeze-dried vaccine. Currently, the general recommendation by the manufacturers is that the opened vaccine vials must be used within six hours. Yet, LSDV is known to be very stable, and, for example, an experimental study has shown that a virulent LSDV kept in phosphate buffered saline (PBS) solution for 35 days at 28 °C was still infective with only some minor loss of titer [32]. More studies are warranted to investigate how long after dissolving the freeze-dried vaccine with a diluent the vaccine solution can be used without losing its efficacy.

Affordability of LSD vaccines is a major obstacle to the use of LSDV-based vaccines for farmers and governments in many developing countries (2). For small-scale cattle owners, it is important that the vaccines are sold in small quantities at a reasonable price. In many LSD-endemic African countries, financial support to cover the LSD vaccines and vaccination by the government is lacking, mainly because LSD is not seen as a high-priority disease due to the low mortality and morbidity rates in endemic settings. The costs of the vaccination are, therefore, covered by farmers themselves. Understandably, smallholders who own only a few animals cannot afford or do not want to buy vaccines in vials that contain more doses than they need. The strict requirement to use the vaccine within six hours after the reconstitution limits the possibility to share the vaccine vials between the farmers. The freeze-dried vaccine is stored at a refrigerator temperature, and for transport, the vaccine vials need to be kept at a stable temperature of 4 to 8 °C. During vaccination field campaigns, it must be considered that all capripoxvirus vaccines are believed to be sensitive to direct sunlight [11] and, therefore, should be kept in the shade. An additional and often overlooked practical challenge is the procurement process of vaccines. The prices of the vaccines vary between different products and depend on the number of ordered dosages. The vaccine price is often the most important, if not the only, vaccine attribute in official tendering processes that may cause delays for the onset of the vaccination campaigns, thus allowing time for the infection to spread. In addition, the vaccines against LSD are not registered or authorized in countries that face the disease for the first time. Moreover, delays may occur at times of high international demand for good quality vaccines, i.e., when the disease is spreading rapidly through new territories, existing producers cannot meet purchase requests. Such logistical or bureaucratic factors may seriously hamper the timely response to an LSD outbreak.

## 3. Live Attenuated Homologous Vaccines

Homologous vaccines provide good protection for cattle against virulent field strains. LSDV vaccines either contain the well-known South African Neethling strain or, despite their confusing names, the KSGP O-240 and O-180 strains. The Neethling strain was isolated from the first LSD outbreaks in South Africa and the vaccine strain was attenuated from the virulent strain by 61 serial passages in lamb kidney (LK) cells, followed by 20 passages in the chorioallantoic membrane of embryonated chicken eggs and three passages in LK cells [31,33]. Another example of attenuation of a virulent field strain is the Madagascan LSD strain, which required 101 passages in rabbit kidney and five in fetal calf kidney cells [31]. Both of these examples demonstrate that a high number of passages are required to attenuate a highly virulent LSDV field strain for the safe to use in cattle. Still, homologous vaccines are known to cause side effects when cattle are immunized for the first time. This can include local skin reaction at the vaccination site or generalized small-size skin nodules and a temporal reduction of the milk yield [13,34], often referred to as “Neethling disease” or “Neethling response”. The good field efficacy of the live attenuated vaccines was demonstrated between 2016 and 2017 when LSD outbreaks in Southeastern Europe were successfully eliminated by coordinated mass vaccination using homologous Neethling strain vaccines [7]. Importantly, the efficacy of the Neethling strain vaccines was experimentally evaluated using a challenge trial by the experts of the European Union LSD reference laboratory at Sciensano in Belgium [35].

Two so-called KSGP strains have been used in cattle against LSD in several countries, such as in Ethiopia [36], Israel [37], and Egypt [38]. Although the name of the strains refers to SPPV and GTPV, the real identity of the Kenyan sheep and goat pox (KSGP) and KS-1 strains has been revealed to be an LSDV strain [29,39,40,41]. There is not much difference between O-240 and O-180 strains as both strains are of ovine origin, isolated during the same outbreak but at different points in time [42,43]. It is obvious, though, that some vaccines might be substantially attenuated in one host species, but too virulent in another [44]. The level of attenuation of KSGP strain vaccines varies generally between five to 30 passages [31], which is low compared to the Neethling strain vaccines. The real identity of the vaccine seed virus (LSD not SPPV/GTPV) explains why the vaccine strain was so easily attenuated for safe use in sheep and goats. However, the low level of attenuation was insufficient for cattle, causing post-vaccination clinical signs, including fever and skin lesions [29]. The minimum recommended field dose of the South African Neethling strain vaccines [45] is log_10_ 3.5 median tissue culture infectious dose (TCID)_50_, although the minimum protective dose is log_10_ 2.0 TCID_50_. Capripoxvirus is highly susceptible to inactivation by sunlight and allowances should be made for loss of activity in the field [28]. According to the manufacturers, all currently available homologous and heterologous vaccines should be administered via subcutaneous route.

## 4. Heterologous Vaccines

### 4.1. Goatpox Virus-Based Vaccines

In 2015, the safety, immunogenicity, and efficacy of a commercially available Gorgan goatpox strain vaccine was, for the first time, evaluated against LSD in Ethiopian cattle, using a combination of a vaccine challenge experiment and the monitoring of the immune responses in vaccinated animals in the field [46]. In this study, the strain provided good protection and seroconversion in cattle against clinical signs of the highly virulent LSD field strain, when used at a volume of one milliliter of a vaccine with the virus titer of either 3.5 or 4.5 TCID_50_/mL. No adverse effects were detected in vaccinated animals. The Gorgan GTPV-vaccinated cattle showed strong cellular immune responses at the vaccination site, measured using delayed-type hypersensitivity (DTH) reactions, indicating high levels of immunogenicity. In another study, the immunogenicity of the Iranian-produced Gorgan GTPV vaccine was compared with the Romanian SPPV vaccine of the same producer in cattle. The seroconversion, Th-1-like IFN-γ and Th-2-like IL-4 cytokine responses were measured and the adverse reactions in vaccinated calves were monitored. Only mild local redness and swelling were detected at the vaccination site without significant statistical differences between the two vaccines. Seroconversion, IFN-γ, and IL-4 levels were higher in animals vaccinated with the GTPV vaccine compared to the SPPV vaccine [47]. In Kazakhstan, two locally produced vaccines based on GTPV (G2-LKW) and Niskhi SPPV strains were tested and compared in cattle. The GTPV strain provided full protection for experimental calves with an average protective index of 5.9 ± 0.0 infectious dose (ID)_50_/0.25 mL, compared to that of the SPPV vaccine group 5.3 ± 1.4 ID_50_/0.25 mL [48]. The GTPV vaccine was grown in LK cells, with an infectious activity of 5.50 ± 0.13 log TCID_50_/mL. The attenuation process comprised 20 serial passages in LK cells. The GTPV vaccines require a lower level of attenuation for safe use in cattle than homologous vaccines. This is a clear benefit compared with LSDV-based vaccines.

The price of the LSDV-based vaccine may not be affordable for countries with large cattle populations. The development of a local vaccine from a GTPV strain may provide a cheaper and faster alternative to commercially available homologous vaccines that need to be obtained from international sources. Recently, India and Bangladesh have used GTPV vaccines against LSD with seemingly satisfactory results [2,49]. Prior to use, the efficacy and safety of any local heterologous vaccine in cattle against LSD need to be thoroughly evaluated to meet the requirements for a good quality vaccine.

### 4.2. Sheeppox Virus-Based Vaccines

Several SPPV based vaccines have been used in cattle against LSD with varying success in Turkey, the Russian Federation, parts of the Middle East, and the Caucasus region. The attenuated Yugoslavian SPPV RM65 strain vaccine was used in cattle in Israel [50] and Jordan [51], while the Romanian SPPV strain in Egypt and Saudi Arabia [52]. The Saudi Arabians immunized their cattle every six months with a ten times stronger dose than that used in sheep [53,54]. However, data and experience obtained from the recent studies and outbreaks indicate only partial cross-protection provided by an attenuated sheeppox vaccine against the LSD field strain [36,37,52,54,55,56]. Incomplete protection was also observed when the Yugoslavian RM 65 (Ramyar) sheeppox vaccine was used to protect cattle from LSD in Israel from 2006 to 2007 [57].

As an initial response to the recent LSD outbreaks, Turkey, Georgia, and Azerbaijan used a Turkish Bakirköy SPPV strain vaccine for the vaccination of cattle in three to ten- times higher dosages compared to that employed in sheep [58]. The veterinary services of the Russian Federation and Armenia chose the Sheep Pox Cultural Dry™ vaccine produced by the Federal Centre for Animal Health (ARRIAH) [58]. The elimination of LSD, using SPPV-based vaccines was not as complete nor as effective when compared to the success of homologous vaccine in the Balkans [7]. Currently, LSD can be considered as endemic in Turkey and possibly in parts of the Russian Federation. Ineffective control of the disease may also be associated with vector transmission, challenges in carrying out the vaccination campaign and controlling cattle movements, as well as the presence of comparatively large cattle population in these countries.

The side effects caused by the SPPV vaccine in naïve cattle are more rarely detected than those caused by attenuated LSD vaccines. However, it has been demonstrated that administration of a high dosage of SPPV RM65 vaccine may produce typical vaccine side effects, such as generalized skin lesions in cattle [59].

### 4.3. Inactivated Vaccines

Inactivated vaccines provide both advantages and disadvantages compared to live attenuated ones. The most important benefit is the safety of inactivated vaccines. Their non-replicative characteristics prevent transmission of the vaccine virus to naïve animals, a reversion to virulence, and recombination with virulent virus strains [60,61]. Inactivated vaccines could provide a safe prophylactic vaccine alternative in disease-free at-risk countries, provided that a robust cattle identification and vaccination/health record system would be in place. However, the OIE Terrestrial Code does not differentiate between live and inactivated LSD vaccines and, therefore, if an at-risk country practices vaccination with any type of vaccine against LSD, it will lose its LSD-free trade status [62]. In addition, the use of an inactivated vaccine would also hamper the serological surveillance, which is one of the criteria set for the demonstration of the freedom of the disease status. In order to establish protection, two initial administrations of the vaccine dose, followed by periodic revaccinations are likely to be required, which would considerably add to the costs and required resources of vaccination campaigns. However, further field experiments are still needed to evaluate the need for booster vaccination for each inactivated vaccine candidate. The need for an appropriate adjuvant to activate the immune system and to improve the immune response in the vaccinated animal enhance the costs of inactivated vaccine products [61,63,64]. Previously, inactivated vaccines against capripoxviruses have been reported to induce insufficient immune responses and to produce only short-lived protection [65], while experiences with smallpox virus vaccines would suggest that a replicating antigen is required [66]. In recent decades, several attempts have been made to generate inactivated vaccines, particularly against SPPV and GTPV, raising hopes for the successful development of inactivated capripoxvirus vaccines. As early as 1982, Sólyom and co-authors published promising results regarding an inactivated SPPV vaccine [67]. Many years later, in 2016, Boumart et al. showed complete protection against clinical signs after strong challenge infections in sheep previously immunized with an inactivated SPPV prototype vaccine [60]. However, it was still several years before similar attempts on inactivated vaccines against LSDV were published.

In 2020, Hamdi et al. and Wolff et al. published their results, describing the efficacy of diverse inactivated LSDV vaccine candidates in comparison to a live vaccine in a cattle vaccine-challenge model [61,68]. In both studies, an attenuated LSDV-“Neethling” vaccine derivative was inactivated with binary ethylenimine and adjuvanted with different adjuvants—an oily emulsion with Montanide adjuvant from SEPPIC [61] and a low molecular weight copolymer (Polygen, MVP Adjuvants^®^, named as Adjuvant A) [68]. Moreover, LSDV-“Neethling” vaccine virus was propagated in primary testis cells [61] and in different permanent cell lines [68], respectively. Cattle were immunized either with the respective inactivated prototype vaccine or with an attenuated live vaccine against LSDV and were challenged with highly virulent LSDV field strains [61,68]. No viremia nor viral shedding via oral fluid was observed in cattle immunized with the inactivated vaccine, but low levels of viral DNA were found in skin samples of a small number of animals with very high C_q_-values [61]. Wolff and co-authors achieved only partial protection against a combined intravenous and subcutaneous challenge infection with LSDV-“Macedonia2016” field strain after vaccination with the inactivated LSDV-“Neethling” vaccine strain. In particular, local reactions at the site of challenge virus inoculation could be observed. Moreover, some animals displayed slight viremia and slight shedding of the virus via ocular, nasal, and oral fluids after challenge infection [68]. In their second study, the same study group compared different inactivated prototype vaccines based on the LSDV-“Serbia” field strain propagated in non-bovine cell lines and formulated with two different adjuvants: the same Adjuvant A as used before and a proprietary adjuvant consisting of a combination of Amphigen, Quil A, and cholesterol (named as Adjuvant B). Furthermore, the challenge model was changed, and challenge infection with LSDV-“Macedonia2016” was performed intravenously [68]. After both primary and secondary immunization, local adverse reactions at the site of vaccination, as well as mild to moderate clinical reaction after challenge infection could be observed in some cattle of those groups vaccinated with vaccine candidates consisting of Adjuvant B. Furthermore, replicable LSD challenge virus could be isolated from the appearing skin nodules after challenge infection, indicating a possible risk for vector-borne spreading of wild-type virus in the field. Interestingly, the Adjuvant A vaccine prototype was completely safe as there were no adverse effects detectable after primary and secondary immunization, and sterile immunity was induced in all vaccinated cattle [68].

When comparing all three studies, conclusions can be drawn regarding the factors that are essential for the successful development of inactivated vaccines against LSDV. Since three different cell culture systems were used, primary cells, a bovine cell line, and a non-bovine cell line, and no differences could be observed between these systems, the influence of the cell culture system on the attenuation process can be considered minor [61,68]. Furthermore, the choice of the LSDV virus strain to be inactivated did not seem to play a major role since both the LSDV-“Neethling” vaccine strain as well as the LSDV-“Serbia” field strain were able to protect cattle from strong challenge infection after administration in inactivated and adjuvanted formulations [61,68]. Using the subcutaneous inoculation of the challenge virus made local reactions more likely [68] compared with intravenous or intradermal inoculations [61,68]. The choice of the adjuvant seems to be of major importance. Adjuvant B induced local adverse reactions after immunization and did not completely prevent clinical symptoms after the challenge infection, thereby leaving the risk of challenge/field virus spread [68]. Whereas, both the oily emulsion with Montanide™ adjuvant from SEPPIC (Air Liquide Healthcare, France) and the low-molecular-weight copolymer were able to induce clinical protection in cattle [61,68]. The latter even induced sterile immunity in all cattle vaccinated and challenged [68]. All used adjuvants delivered a strong humoral immunity, but in the case of Adjuvant B, this robust humoral response was insufficient for complete clinical protection [68]. This supports the assumption that antibody-independent immune reactions are necessary to deliver full and sustainable protection [47,69,70]. Although both studies show promising results with inactivated LSDV vaccine prototype candidates, several questions remain unanswered. In particular, the minimum protective dose and the duration of immunity warrant further investigations. More research is needed to evaluate the (cellular) factors for a robust and lasting immunity after vaccination with inactivated capripoxvirus. However, in combination with novel and appropriate adjuvants, the implementation of an inactivated vaccine against LSDV seems to have become achievable.

## 5. Seroconversion and Duration of Immunity after Vaccination with LSD Vaccines

Immunity against poxviruses is both humoral and cell mediated [71]. Animals that recovered from natural LSDV infection developed antibodies capable of neutralizing up to three-log TCID_50_/mL of the virus and were resistant to reinfection [11]. Antibodies are believed to play an important role during the early stages post-infection. Seroconversion measured using either serum/virus neutralization test or enzyme-linked immunosorbent assay (ELISA) method starts approximately 10–15 days after vaccination and reaches the peak levels about 30 days post-vaccination, after which titers gradually decline to undetectable levels. Yet, the susceptibility of previously infected or vaccinated animals cannot be directly related to serum levels of neutralizing antibodies. It is known that not all vaccinated animals seroconvert, although they are fully protected against LSD [11,72,73]. Measuring anti-LSD antibodies alone may, therefore, not provide sufficient data on the protection status of the vaccinated animals, which must be considered when the effectiveness of the vaccination campaigns are evaluated in vaccinated herds. As an example, in a study by Milovanovic and co-workers, only 33% (*n* = 26/79) of vaccinated cattle remained ELISA-positive 11 months after the first vaccination. A booster vaccination was given 12 months after the initial vaccine. Five months after the booster vaccination, 57% (*n* = 42/74) of the vaccinated animals remained positive. Approximately 27% (*n* = 21/79) of cattle remained seronegative after both the initial and the second vaccinations [73]. Nevertheless, this study did not include a challenge trial post-vaccination and, thus, insufficient protection for the antibody-negative animals cannot be excluded. Similar findings have been reported after the use of smallpox vaccine; some vaccinated people were so-called low responders, producing only low levels of antibodies [74], which may not be detectable with serological methods. This has been associated with genetic differences between individuals and up and downgrading of genes [75]. After the antibody levels have declined beyond the detectable level, the cell-mediated immunity still protects the vaccinated animals. Post-vaccination immunity is likely to persist for two to three years, although the exact duration of the immunity provided by any currently available vaccine still needs to be experimentally demonstrated. Mainly for practical or safety reasons, the vaccine manufacturers recommend annual vaccinations. Regarding sero-surveillance, the presence of low responders in the vaccinated or naturally infected herds should be considered if the evaluation of the immune status is based solely on serology; positive cattle are protected but negative animals may or may not be protected. Calves born to immunized cows will have a passive immunity that persists for about three to four months [76]. In 88.9% (*n* = 16/18) of calves receiving colostrum from vaccinated dams, neutralizing antibodies were detected in their serum three days after birth. The levels of antibodies started to decline with time—after 90 days, only 38.5% (*n* = 5) were positive, and after 120 and 150 days, none of the calves had detectable antibodies [76]. In another study, only in 10% (*n* = 3/30) of the calves maternal antibodies were detectable after three months [77].

Despite the above-described challenges, sero-surveillance is often required for the demonstration of freedom of the disease. All capripoxviruses cross-react serologically and serology does not provide tools to differentiate if the animals are vaccinated using LSDV, SPPV, or GTPV strain vaccines. Recently, a commercially available ELISA by ID Innovative Diagnostics (Montpellier, France) has been demonstrated to be suitable for the detection of antibodies in serum and individual and bulk milk samples [78]. How long after vaccination or natural infection the antibodies can be detected in the blood samples is subject to individual variation. More detailed data on seroconversion in vaccinated animals are urgently needed to provide feasible guidelines and tools for countries that need to carry out sero-surveillance, either during or after an outbreak, or those who wish to monitor the efficacy of their vaccination campaigns by using serological studies.

## 6. Experimental Evaluation of the Efficacy of a Vaccine and Vaccination Effectiveness Studies

A challenge model for the evaluation of the efficacy of a vaccine against LSD has been described previously [35,79]. A minimum of six to eight healthy animals should be included in a challenge trial and an additional five to six animals should serve as unvaccinated control. Most often, calves at the age of six to twelve months are used as experimental animals for practical reasons. In that age group, both male and female calves can be included. Prior to the onset of the experiment, cattle need to be tested to be seronegative and free of ongoing LSD infection. Acclimatization to the controlled environment of one or two weeks is required to avoid the effect of stress-related factors. Vaccination should be undertaken strictly according to manufacturers’ recommendations. Three to four weeks post-vaccination, vaccinated and control groups are infected with a highly virulent LSDV field strain (wild-type). Experiences obtained from previous animal experiments indicate that in order to produce visible clinical disease in half of the susceptible experimental cattle, the titer of the challenge virus needs to be between 10^4.0^ to 10^6.5^ TCID_50_. Several different cells can be used to grow the virulent field strain. The application of the challenge virus via the intravenous route is important and can be combined with a local inoculation via the intradermal or subcutaneous route. After the challenge, the animals should be closely monitored and the findings recorded for up to four weeks for the appearance of clinical signs, such as fever, nasal and eye discharge, excessive salivation, enlarged lymph nodes, local or generalized skin nodules, and lesions in the muzzle and/or mucous membranes of the mouth and nasal cavities. Every second day, samples should be collected for PCR and virus isolation. EDTA blood, as well as saliva and nasal swabs are the recommended sample material to analyze viremia as well as virus excretion. In addition, biopsies from the typical skin nodules can be tested with polymerase chain reaction (PCR) and used for the virus isolation purpose. Molecular tools have been described to differentiate a vaccine from a field virus [68,80,81,82,83].

A retrospective surveillance of vaccine effectiveness not only measures the efficacy of the vaccine, but also the implementation of the vaccination campaign. Active clinical surveillance is a good tool when combined with serological surveillance. The reliability of the clinical surveillance and sero-surveillance heavily builds on the ability to know which animals are vaccinated and which ones are not. If no animal health record databases/systems are available, a permanent way to mark the vaccinated animals should be developed for the purpose. During outbreaks with a virulent virus circulating in the region, it should be considered that it takes two to three weeks until the vaccine provides full protection, before that animals may still become infected and exhibit clinical signs typical for LSD.

Serological surveys after vaccination campaigns are complicated by the fact that some vaccinated animals, and those individuals showing a mild disease, may develop only low antibody levels although they are fully protected [11,84]. The unclear meaning of seronegative animals decreases the value of sero-surveillance as a sole method. Seronegative animals in a vaccinated herd may or may not be protected by the vaccine or they may have been missed during the vaccination campaign. The most reliable results are obtained when the serum samples are collected not earlier than one month and not later than five months after vaccination [73]. The sample size calculations need to take into consideration the percentage of animals that are expected not to develop any detectable antibody levels after vaccination. Passive surveillance provides an additional tool if the awareness levels regarding LSD are high amongst cattle farming stakeholders and if farmers are willing to report. Commercially available pan-capripoxvirus ELISA is available for large-scale testing, allowing easier monitoring of the duration of humoral responses in vaccinated herds than using a serum neutralization method.

Currently, there are no DIVA (differentiating infected from vaccinated animals) vaccines with associated marker tests commercially available for LSDV. Therefore, it is not possible to differentiate between vaccinated and naturally infected animals based on serology. Even if a DIVA strategy would be available, it is questionable whether it would be affordable or feasible for low-resource countries, most likely due to the lack of animal identification/vaccination/recording systems and the extra costs and resources required by the surveillance programs. Measuring the cytokine (e.g., IFN-γ) response, such as in blood samples collected from vaccinated animals, could potentially provide an additional diagnostic tool in the future. Currently, standard operating procedures and commercially available tests validated for this approach are still missing.

## 7. Vaccine Side Effects and Safety

The safety of live homologous vaccines in previously LSD-free countries is often of major concern. According to vaccine producers, the development of protective immunity takes approximately two to three weeks post-vaccination. During this time, animals can still show clinical signs if they become infected by a wild-type LSD virus. Usually, adverse reactions appear about one to two weeks after vaccination and often comprise a local reaction at the vaccination site and, more rarely, generalized skin lesions, known as a “Neethling response” [35]. A temporary decrease in milk production may occur in vaccinated animals [13]. The level of attenuation of the vaccine product has a major effect on the appearance of fever and local or generalized skin reactions after vaccination with homologous vaccines. The skin lesions caused by the vaccine strain are clearly smaller and can easily be differentiated from those caused by virulent field strains [34]. SPPV and GTPV vaccines do not usually cause any adverse effects in cattle. However, when a high dose of a vaccine based on the RM65 SPPV strain was used in cattle, some mild adverse reactions were reported [59]. Field experience obtained on the use of LSDV vaccines in the Balkan region and elsewhere indicated that the homologous vaccines caused side effects only when used for the first time in a previously disease-free country. The booster vaccination does not inflict reverse reactions, even if the initially used vaccine was a heterologous vaccine [34].

According to a study from Israel, a homologous LSDV-based vaccine caused only mild adverse effects at very low incidence (0.4%, *n* = 9/2356) [50]. However, in the same study, the cattle cohort was already pre-vaccinated with a SPP RM65 vaccine strain, which likely reduced the observed number of adverse effects [50]. In a follow-up study, by the same authors, daily milk production data, as well as statistics on culling and mortality, were retrieved from 21,844 cows in 77 dairy cattle farms. Adjusted milk production was calculated daily for 30 days post-vaccination and compared to the preceding month by fitting mixed-effects linear models. Culling and mortality rates were compared between the 60 days prior and post-vaccination via survival analysis. According to the results of the models, no significant change in milk production was detected during the monitoring period (30 days post-vaccination). In addition, there was no difference in routine culling between the pre- and post-vaccination periods or in immediate culling and mortality [85]. In the Balkan region, despite the annual vaccination of 1.8 million cattle with a live LSD vaccine [7], no outbreaks caused by the vaccine strain were reported. Croatian scientists isolated the LSD vaccine virus strain from some vaccinated animals in blood, skin lesions, milk, and saliva [86]. In another study, the sequencing of the whole genome of a LSDV isolate, collected from skin samples of a vaccinated animal, demonstrated that after a passage in cattle, the genome of the vaccine virus remained unchanged and fully attenuated with 100% sequence homology to the original vaccine virus [87].

Freedom from extraneous viruses is crucial for a safe vaccine. If homologous or heterologous vaccines against LSDV are propagated on ovine or caprine primary cells, the presence of pestiviruses (bovine viral diarrhea and border disease), bluetongue virus, foot and mouth disease virus, and rabies virus must be ruled out in each cell batch.

Recombination of poxviruses is believed to be an infrequent event and published examples of poxvirus recombination in the field have been scarce, except in some laboratory conditions [7]. However, by comparing the detailed physical maps of four capripoxviruses, Gershon et al. 1989 were able to show recombination of some parts of the genome of Yemen goatpox-1 isolate, derived from Iraqi goat-1 with the genome of KS-1 strain [88], highlighting an example of recombination during a natural virus transmission. Such recombination can occur if an animal, harboring a virulent field strain, is vaccinated with an insufficiently attenuated live vaccine. The recently published sequencing data of two recombinant LSDV strains suggested a backbone from the Neethling-like vaccine virus and pieces of wild-type virus, resembling the KSGP vaccine [30,89,90]. In 2017, the first recombinant LSDV Russia/Saratov/2017 was isolated from a bovine of a backyard farm in Saratov, close to the border with Kazakhstan, by Russian scientists. Later, the same research group provided experimental data on the virulence of this isolate [19] and also identified the second recombinant virus, LSDV Russia/Udmurtiya/2019, genetically close but still different from the first one. The study on the Russian recombinant LSDVs pointed out the potential role of illegal use of LSDV vaccines. This hypothesis is consistent with Gershon et al.’s 1989 study, which highlighted that recombination could be a possible outcome arising from the use of live under-attenuated poxvirus vaccines [88].

Some manufactures in LSDV-endemic countries produce both KS1 and Neethling vaccines, increasing the risk of cross-contamination between the two vaccine viruses. If a vaccine is composed of a mixture of viruses, they may recombine during the cell culture process. Indeed, there are many examples of successful reproduction of recombination under laboratory conditions in vitro [91,92,93,94]. Such recombination, taking place in a vaccine product composed of multiple variants of the same virus was demonstrated for the Dryvax vaccine [95]. Either way, these findings further highlight the danger of using under-attenuated homologous or heterologous vaccines for cattle during an outbreak.

Another Russian research group pointed out the use of SPPV vaccine as an enabling factor that favored the emergence of multiple LSDV strains in previously vaccinated animals in the Saratov region [96]. The same authors argued that following the use of homologous vaccine in the region, the incidence of LSDV in vaccinated animal declined [96]. It is crucial to adopt multi-target procedures, exploiting genes located at various positions across the genome, to analyze LSDV strains and differentiate the vaccine strain from field viruses. Furthermore, the availability of molecular tools for easy screening of the viral population in LSDV vaccines is urgently needed. It is extremely important to test the efficacy of commercially available vaccines against these novel recombinant strains isolated from clinical cases in the field. This would allow for a better understanding and evaluation of the role of the recombinant LSDV strains during outbreaks.

## 8. The Role of Vaccination in LSD Prevention and Control

### 8.1. Strategic Considerations in LSD Vaccination

Vaccination represents the core tool in efforts to prevent and control LSD, yet its application varies greatly around the globe, not only due to the different epidemiological settings but also due to the socio-economic background and the pursued strategic goals in its implementation. A feasible vaccination approach depends on the epidemiological perspective, whether the country is facing an epidemic of LSD or the disease is already endemic. Alternatively, the country may face diverse at-risk scenarios. For all concerted vaccination efforts, a sound animal identification system will play a crucial role when monitoring the chosen vaccination strategy and control or elimination programs. In most cases, the efforts associated with the implementation of such an identification system will only pay off if they are part of multi-disease control or elimination programs [97]. If such a system is not in place, a minimum requirement is to mark the vaccinated animals permanently (e.g., vaccination-campaign-specific ear tags or markings). In an endemic environment without other organized approaches to control the disease, farmers may simply perform vaccination as a stand-alone protection measure. For many areas in sub-Saharan Africa, this has been the reality over the past decades [98,99,100], highlighting the need to adapt vaccination strategies [101]. Facing localized LSD outbreaks, countries have opted for ring vaccinations of cattle and Asian water buffalo populations surrounding the outbreak(s) [7,98]. In combination with rigorous movement restrictions, this approach can succeed, yet due to the vector-borne local spread patterns, the decision on the size of the vaccination area (older examples state 25 to 50 km radius) must be carefully re-evaluated [98], considering the overall consequences for surveillance and control [102]. Countries successfully pushing back a recent LSD introduction, or preventing LSD incursions, have opted for vaccination of the susceptible population for several years, as seen in southeastern Europe and Israel [1]. It has to be noted, however, that when conducting a shift away from a compulsory vaccination scheme, additional emphasis on the disease surveillance programs is required in order to avoid disease recrudescence [7]. Sufficient vaccination coverage (80–100%) is essential for the success of disease control. In practice, all bovines, including domestic buffalos, need to be immunized and, ideally, an updated animal database should be in place that includes data on animal ID, vaccination details, health records, and a full animal movement history. Vaccine manufacturers recommend annual vaccination of bovines. Nevertheless, it can be expected that appropriately vaccinated cattle will produce a long-lasting immunity after the first, or at least after the second, vaccination. Consequently, the immunization of naïve animals could be considered more important than the re-vaccination of cattle. In the view of limited resources for a vaccination campaign, this targeted immunization strategy can be an advantage for the control of LSD. Faced with an outbreak, calves from unvaccinated dams can be vaccinated at any age. In general, calves from vaccinated or naturally infected mothers should be vaccinated at the age of three to four months, when maternal antibodies are declining or already absent [76]. No LSD vaccine challenge experiments have been carried out in domestic buffaloes, but the vaccination protocol using the same dosage and protocol for cattle has been recommended [25]. Newly purchased animals, or cattle intended to be moved, should be vaccinated at least 28 days before transport. According to vaccine manufacturers’ recommendations, only healthy cattle should be vaccinated with a live vaccine. Pregnant, healthy cows and heifers can be safely vaccinated. Vaccinated breeding bulls did not excrete vaccine virus to semen. Moreover, vaccination prevented the excretion of LSD virus in semen after these bulls were challenged [103].

### 8.2. Vaccination-Oriented Risk Assessment

For maximum impact, vaccination against LSD should be embedded into existing disease control programs and risk-based surveillance, rather than stunting potential vaccine efficacy by treating it as a stand-alone measure [104,105]. The design of such disease control programs must consider the decision to vaccinate, economic effects of vaccination, the choice and availability of vaccine types, the timing and spatial extent of vaccination, the monitoring of the disease and vaccination progress, as well as the synergistic combination with additional, locally feasible control measures. To this end, epidemiologically founded risk assessments for the incursion and spread of LSD help to establish adequate LSD control programs and to form a critical basis for sound vaccination strategy planning, along with information provided in this article. Recently published risk assessment frameworks for LSD incursion mostly focus on currently disease-free countries, such as Scotland [106], the UK as a whole [107,108], Germany [109], and France, and some already infected countries, such as Turkey [22], through movements of cattle [110] or vectors that may transmit LSD [110]. More spatially explicit LSD risk assessments identify regions with a high risk of LSD occurrence at much finer geographic scales [111] or even at individual farm-level [112], thus justifying and guiding control-program-embedded vaccination efforts. It is advisable to review risk assessments for LSD vaccination planning on a regular basis to accommodate new LSD vaccine developments [68] and unforeseen changes in the epidemiological disease situation. LSD risk factors that are typically evaluated for risk assessment purposes are often poorly understood. These factors should include the following risk categories: Environmental factors, such as climate and geomorphology, potentially influence arthropod vector biology [113,114,115], husbandry and biosecurity practices [24,116,117], cattle characteristics and their immune status [76,117,118,119], regulatory disease control and surveillance structures [104,120], animal- or personnel-associated movements [107,108,110,121,122], as well as societal factors [2,23,123]. Risk assessments and control of the risk factors for LSD offer valuable support in scenarios where LSD vaccination is not desired or possible for regulatory, economical, or infrastructural reasons, thus shifting from vaccination-centered disease control programs to strengthening LSD biosecurity, awareness, relevant movement regulations, and risk-based surveillance. In summary, risk assessments guide LSD vaccination and control strategies by integrating current knowledge of disease control strategies, epidemiology, risk factors, and transmission dynamics. However, to date, no country has been able to eliminate the disease from its territory without vaccination.

## 9. Development of Future Vaccines

There are no DIVA or subunit vaccines, including their associated marker tests (e.g., based on serology), available against LSD. Experimentally, LSDV has been successfully used as a backbone for several recombinant subunit vaccines, such as rabies [124], rinderpest [125,126], Rift Valley fever [127], and HIV [128,129]. From the scientific point of view, application of a DIVA vaccination strategy would be the optimal to control and monitor the spreading of LSDV in a country or region. Nevertheless, some substantial disadvantages of the DIVA strategy exist and minimize the general acceptance. A LSDV-DIVA vaccine strain should be easily available and producible for all users without significant additional costs. In addition, a validated serological test for the differentiation of antibodies from field infection and vaccination must be available and easily applicable. Furthermore, all advantages of a DIVA strategy can only be realized if an animal identification/recording system can be used and applied. It is in generally questionable whether the benefits of a DIVA strategy justify the additional effort and costs involved, especially in low-resource countries.

## 10. Challenges with Transboundary LSD in Low-Income Countries and One Health Capacity Building

Over the past few years, LSD has seen an unprecedentedly wide geographic spread from Africa to the Middle East, southeast Europe, and Asia (Figure 1). The risk of LSDV incursion is particularly high in areas that share common borders with infected countries. Both officially authorized and illegal movements and trade of bovines increase the risks of spreading the disease. In newly infected countries, the level of general and technical awareness on LSD is usually low amongst veterinarians, laboratory experts, farmers, and others along the value chain. International organizations play a key role in providing assistance, training, coordination, and regional harmonization of disease control measures, improving laboratory capacities and designing vaccination strategies and post-outbreak exit plans. All these actions will contribute to better preparedness of countries to combat LSD and ensure safe trade in livestock. Of particular relevance is the need to provide guidance on the selection of the right vaccine and vaccination strategies. Notifiable animal disease databases such as WAHIS and EMPRES-i allow close monitoring of the global disease situation, which feeds directly into continuous risk assessments and associated trade recommendations for cattle and cattle products [2,130,131]. In addition, the Food and Agricultural Organization of the United Nations (FAO) offers online tools free of charge to estimate the cost of LSD outbreaks and to map areas at risk of LSD spread [2,12,111]. Online webinars and virtual training platforms provide effective means to connect veterinary authorities, decision makers, official veterinarians, and private field veterinarians with disease experts around the globe. In 2020/2021, in response to the aggressive spread of LSDV, FAO and The European Commission for the Foot-and-Mouth Disease (EuFMD) organized a four-week tutored virtual training course on LSD, allowing the training of thousands of veterinarians in Europe, Asia, and Africa. The training modules were also adapted to Asian, ex-Soviet Union, and African cattle farming practices and delivered to participants from those regions. Face-to-face training is still valuable via national and regional workshops. The FAO provides guidelines, manuals, templates for contingency plans, and awareness materials for LSD [25,58,132]. A recent example of successful regional coordination was the elimination of LSDV circulation in the Balkans, following its introduction in 2015/2016. Control efforts were largely coordinated through the Global Framework for the Progressive Control of Transboundary Animal Diseases (GF-TADs), a joint initiative of the FAO and OIE, together with the European Commission to prevent and control TADs while addressing their regional or global dimensions.

Due to the vector-mediated mode of transmission of LSDV and the practical difficulty of restricting the local and transboundary movement of bovines, the control and eradication of LSD is highly dependent on the chosen vaccination strategy. Good vaccines against LSD are already commercially available. Thorough preparedness and the acquisition of expertise on LSD will improve the ability of veterinary authorities to make timely decisions on vaccines and vaccination-related issues, which will determine the spread of the disease and the magnitude of the economic losses to the cattle farming industry as a whole. Regional coordination of the disease control is necessary. National eradication of LSD would be an enormous challenge if the surrounding countries remain infected. In contrast, regional eradication of LSD would be a more achievable goal if international organizations and national governments were strongly committed to its control and if farmers supported associated measures.

Complete eradication of LSD requires disease awareness, transparency in disease notification, regionally harmonized control/eradication policies and viable surveillance programs in affected and at-risk areas. Adequate funding must be available for human resources, laboratory testing, and vaccines. Moving forward, a simple but reliable system to distinguish vaccinated from unvaccinated animals and well-designed regional surveillance programs to demonstrate “freedom from disease” status would also be essential.

In the developing world, the social and financial importance of cattle keeping is unique and has a wide-scale effect on the rural economy as a whole: it provides income for farmers, improves the health and nutrition of the human population, allows children to go to school, and produces traction power and fertilizer for growing crops. Healthy livestock means wellbeing and growth for rural communities, sustainable use of natural resources, and a healthier environment. LSD has a substantial negative effect on cattle production but, in addition, it increases the use of broad-spectrum antibiotics in cattle [133] and the large-scale use of insecticides on animals and in the environment in order to reduce the number of vectors. Due to LSD, the risk of developing antibiotic resistance against human and animal pathogens is increasing [134]. Insecticides sprayed into the environment also kill useful insects, such as pollinators, and, thus, negatively affect biodiversity. Currently, the One Health concept is often perceived to cover mainly zoonotic viruses, but widening the scope to transboundary, high-impact animal diseases with a direct or indirect effect on human and environmental health should be considered. Diseases at the human–animal–ecosystems interface are increasingly addressed following the One Health approach, which recognizes the health of animals, humans, and the environment as inevitably linked to each other. The call for transdisciplinary collaboration considers public health problems from a more holistic perspective, accounting for diverse factors and conditions that shape the way people and animals live together [135]. A One Health approach should take various stakeholders and aspects into account beyond just medical health (e.g., socio-economic, cultural, environmental), foster cross-sectoral collaboration, and break down silos when considering diseases. The expected benefits over a one-disease strategy would include improvements for the whole health system. In the sense of One Health, animal health interventions, even for a non-zoonotic disease like LSD, may yield human health benefits and stronger communities through increased economic savings and livestock productivity, e.g., for nutritional purposes that would be at risk without interventions such as vaccination. Benefits from directly integrating human and animal health interventions have also been described—In Chad, Mali, and Mauritania, a transdisciplinary approach allowed using the capacity of existing veterinary infrastructure for human and animal vaccination [136,137]. Tackling, simultaneously, a number of livestock diseases or animal/human health issues through integrated control packages seems economically and logistically beneficial, even if not all of the diseases are zoonotic. For the most vulnerable rural communities, healthy livestock equals better and more sustainable livelihoods and wellbeing, which aligns with the United Nations Sustainable Development Goals (SDGs) of reduced poverty, zero hunger, and improved human health and wellbeing (https://sdgs.un.org/goals, last visited 23 September 2021).

Sustainable eradication of LSD is an enormous challenge in countries that are neighboring LDS-infected regions. Success of disease control is highly dependent on the chosen vaccination strategy. Eradicating LSD regionally is challenging but achievable, as the example of the Balkans shows. The GF-TADs offers a platform to coordinate regional cooperation of governments and veterinary services. Technical support and assistance can be provided for newly affected countries. Efforts are also required to gain the confidence of the farmers and their commitment for the cause. Comprehensive vaccination coverage combined with high disease awareness, transparency in disease reporting, competence and sufficient resources of local reference laboratories to carry out diagnostics, harmonized control policies, and a viable surveillance program in affected and high-risk areas are the cornerstones of LSD eradication. These key elements are based on a simple but reliable system to distinguish between vaccinated and unvaccinated animals. The future will show whether the economic impact of LSD outbreaks is considered large enough to justify adequate regional multiannual funding programs to finance vaccination strategies, human and laboratory resources, and surveillance programs, or whether LSD will eventually be accepted as one of the endemic livestock diseases that farmers will have to live with.

## Figures and Tables

**Table 1:** Most commonly used vaccines registered for use in cattle against lumpy skin disease (LSD). (Links accessed on 22 September 2021).

Manufacturer	Product Name and Virus Strain	Target Species	Titre, Dose, Administration	Presentation Doses/Vial
**Onderstepoort Biological Products (OBP)** South Africa Email: info@obpvaccines.co.za http://www.obpvaccines.co.za (accessed on 22 September 2021)	Lumpy Skin Disease Vaccine for Cattle (LSD Neethling strain)	Cattle	Not known 2 ml SC	25/50
**Intervet (Pty) South Africa/MSD Animal Health**http://www.msd-animal-health.co.za (accessed on 29 September 2021)	Lumpyvax™ (LSD SIS Neethling type strain)	Cattle	10^4.0^TCID_50_/dose 1 ml SC	20/100
**MCI Santé Animale** Morocco Email: contact@mci-santeanimale.com http://www.mci-santeanimale.com/en/ (accessed on 29 September 2021)	Bovivax-LSD™ (LSD Neethling strain)	Cattle	10^3.5^TCID_50_/dose 2 ml SC	25/50/100
**Jordan Bio-Industries Center (JOVAC)** Jordan Email: sales@jovaccenter.com http://www.jovaccenter.com (accessed on 29 September 2021)	LumpyShield-N™ (LSD Neethling strain)	Cattle	10^4.0^TCID_50_/dose 1 ml SC	5/10/25/50/100
**Middle East for Vaccines (MEVAC)** Egypt Email: marketing@me_vac.com https://www.me-vac.com/about (accessed on 29 September 2021)	MEVAC LSD (LSD Neethling strain)	Cattle	10^3.5^TCID_50_/dose 1 ml SC	10/25/50
**National Veterinary Institute (NVI)** Ethiopia Email: nvi-rt@ethionet.et	Lumpy Skin Disease Vaccine (LSD Neethling strain)	Cattle	10^3.0^TCID_50_/dose 1 ml SC	5/20/100
**Kenya Veterinary Vaccines Production Institute (KEVEVAPI)** http://www.kevevapi.org/ (accessed on 29 September 2021)	Lumpivax™ (Live attenuated LSDV)	Cattle	TCID_50_ not known 2 ml SC	50/100/150
**Pendik Veterinary Control Institute/** **Ministry of Agriculture,** Turkey	Penpox-M™ Live SPPV (Bakirköy SPPV strain)	Cattle	10^2.5^TCID_50_/dose 3 ml SC	
**Vetal Company** Turkey Email: vetal@vetal.com.tr http://www.vetal.com.tr (accessed on 29 September 2021)	Poxvac™ (Bakirköy SPPV strain) Lumpyvac™ (LSD Neethling strain)	Sheep Cattle Cattle	10^2.5^TCID_50_/dose 3ml SC 10^3.5^TCID_50_/dose 2 ml SC	20/50/100/200 10/25/50/100
**Dollvet** Turkey Email: dollvet@dollvet.com.tr http://www.dollvet.com.tr (accessed on 29 September 2021)	Poxdoll™ (Bakirköy SPPV strain) LSD-NDOLL (LSD Neethling strain)	Cattle Sheep Goats Cattle	10^2.5^TCID_50_/dose 3ml SC 10^3.5^TCID_50_/dose3ml SC	50/100 10/25/50/100
**FGBI-Federal Centre for Animal Health**, The Russian Federation Email: mail@arriah.ru http://www.arriah.ru (accessed on 29 September 2021)	Sheep Pox Cultyral Dry™(Arriah SPPV strain)	Sheep Cattle	Not known	50/100

## Data Availability

Not applicable.
